# *Staphylococcus aureus* Colonization and Long-Term Risk for Death, United States

**DOI:** 10.3201/eid2211.160220

**Published:** 2016-11

**Authors:** Angelico Mendy, Edgar R. Vieira, Ahmed N. Albatineh, Janvier Gasana

**Affiliations:** University of Iowa, Iowa City, Iowa, USA (A. Mendy);; Florida International University, Miami, Florida, USA (E.R. Vieira, J. Gasana);; Kuwait University, Kuwait City, Kuwait (A.N. Albatineh)

**Keywords:** Staphylococcus aureus, MSSA, MRSA, methicillin resistance, survival, mortality, prognosis, colonization, antimicrobial resistance, bacteria, United States

## Abstract

To examine the association of colonization by *Staphylococcus aureus* and general population mortality, we followed 10,598 adults for 8.5 years on average. Methicillin-susceptible *S. aureus* colonization was not associated with death. Methicillin-resistant *S. aureus* carriage predicted death in a crude analysis but not after adjustment for socioeconomic status and co-morbidities.

*Staphylococcus aureus* is a common cause of mild to life-threatening infections. It is differentiated into methicillin-susceptible *S. aureus* (MSSA) and methicillin-resistant *S. aureus* (MRSA); the emergence of isolates resistant to vancomycin has raised concern that the bacterium might become untreatable with current antimicrobial drugs (*1*). Whether *S. aureus* colonization influences general population mortality remains unclear (*2*,*3*). Therefore, we examined the association in a US population representative sample followed for nearly a decade.

## The Study

We used data from the 2001–2004 National Health and Nutrition Examination Survey (NHANES) and the NHANES 2001–2004 Mortality-Linked File. NHANES is a survey of the US noninstitutionalized civilian population conducted by the Centers for Disease Control and Prevention’s National Center for Health Statistics. We selected 10,598 NHANES participants using a complex multistage sampling design and followed them through December 31, 2011, to track mortality. NHANES protocols were approved by the institutional review boards of NCHS and CDC, and all participants provided informed consent.

Nasal swabs collected from participants were tested for *S. aureus* and methicillin resistance during 2001–2004 following the National Committee for Clinical Laboratory Standards disk-diffusion method. We additionally tested isolates resistant to oxacillin (MRSA) and intermediately resistant to oxacillin and every tenth isolate sensitive to oxacillin (MSSA) for staphylococcal cassette chromosome *mec* (SCC*mec*) typing, genes encoding enterotoxins, toxic shock syndrome toxin-1, and Panton-Valentine leukocidin (PVL) toxin (details on the methods are available at http://www.cdc.gov/nchs/data/nhanes/nhanes_01_02/l35_b_doc.pdf). We collected data on age, sex, race/ethnicity, family income, smoking, and medical conditions using questionnaires.

Univariate and multivariate Cox proportional hazards regressions were used to estimate the hazard ratio (HR) with corresponding 95% CIs for MSSA and MRSA association with mortality. Analyses were performed in STATA version 11 (StataCorp LP, College Station, TX, USA), taking into account the survey design and sampling weights, to produce nationally representative estimates. We considered p values <0.05 as statistically significant.

A total of 27.3% of participants were colonized with MSSA and 1.3% with MRSA (Table 1). During an average of 8.5 years of follow-up, the total death rate per 1,000 person-years was 10.3: 10.8 for noncolonized participants, 8.8 for those colonized with MSSA, and 25.6%for those colonized with MRSA. MRSA-colonized participants tended to be older and non-Hispanic white, have low poverty income ratio, smoke cigarettes, have co-morbidities, or have been be hospitalized and/or have had dialysis in the previous 12 months (Table 1). Approximately 58.6% of MRSA isolates were of SCC*mec* type II, suggestive of hospital-associated MRSA, and 41.4% had MRSA isolates of SCC*mec* type IV, indicative of community-associated MRSA.

**Table 1:** Characteristics of study participants by *Staphylococcus aureus* colonization status, National Health and Nutrition Examination Survey, United States, 2001–2004*

Characteristic	No *S. aureus*	MSSA	MRSA	p value†	All participants, N = 10,598
Prevalence	71.4	27.3	1.3		100
Median age, y (IQR)	43 (31–56)	41 (30–54)	54 (34–67)		43 (31–55)
Sex				<0.001	
M	46.1	54.0	36.4		48.1
F	53.9	46.0	63.6		51.9
Race/ethnicity				<0.001	
Non-Hispanic white	70.3	74.4	80.8		71.5
Non-Hispanic black	12.1	8.4	11.7		11.1
Hispanic	12.4	12.8	4.5		12.4
Other	5.2	4.5	3.0		5.0
Body mass index				.28	
Normal or underweight	34.6	32.6	33.8		34.0
Overweight or obese	62.4	65.0	63.6		63.2
Missing	3.0	2.4	2.5		2.8
Poverty income ratio				<0.001	
<1.85	30.4	29.4	42.7		30.3
>1.85	64.1	64.3	47.7		63.9
Missing	5.5	6.3	0.6		5.8
Smoking	49.2	44.6	63.8	<0.001	48.1
Co-morbidities					
Asthma	11.9	12.9	20.5	.07	12.3
Diabetes	8.1	9.4	13.5	.13	8.6
Hypertension	33.0	30.3	39.5	.05	32.4
Cardiovascular diseases‡	6.1	4.6	12.5	.01	5.7
Stroke	2.7	1.9	4.9	.03	2.5
Liver disease	3.4	3.1	2.6	.76	3.3
Cancer	8.9	7.3	16.7	.02	8.5
Hospitalization and/or dialysis in past 12 mo	10.5	10.6	19.8	<0.001	10.6
Mean survival, y (SE)	8.47 (0.11)	8.67 (0.10)	7.5 (0.28)	<0.001	8.52 (0.10)
Death during follow up	9.1	7.7	19.3	<0.001	8.9

*All values are percentages except as indicated. IQR, interquartile range; MSSA, methicillin-susceptible *S. aureus*; MRSA, methicillin resistant *S. aureus*.  †p values for comparison of non–*S. aureus* colonization vs. MRSA colonization vs. MSSA colonization; p values calculated using χ^2^ test for categorical variables and analysis of variance for continuous variables. ‡Coronary heart disease, congestive heart failure, and myocardial infarction.

In Cox regression, MSSA-colonized participants had a significantly lower risk for death than did noncolonized participants in univariate analysis (hazard ratio [HR] 0.82, 95% CI 0.68–0.98) but not after adjustment for co-variates. MRSA colonization was associated with higher risk for death than no *S. aureus* colonization in univariate analysis (HR 2.40, 95% CI 1.56–3.69) and after adjustment for sociodemographic factors (HR 1.54, 95% CI 1.06–2.34); MRSA colonization also carried higher risk for death than MSSA colonization (HR 2.90, 95% CI 1.92–4.40). However, after additional adjustment for smoking, body mass index, and co-morbidities (asthma, diabetes, hypertension, cardiovascular diseases, stroke, liver disease, and cancer), these associations were no longer significant (Table 2).

**Table 2:** Cox proportional hazards regression for *Staphylococcus aureus* colonization and long-term mortality, United States*

Exposure variable‡	No. deaths	No. patients	Unadjusted			Model 1			Model 2	
			HR† (95% CI)	p value		HR† (95% CI)§	p value		HR† (95% CI)¶	p value
*S. aureus* colonization										
No *S. aureus*	1,051	7,794	1 (referent)			1 (referent)			1 (referent)	
MSSA	279	2,668	0.82 (0.68–0.98)	0.035		1.03 (0.85–1.24)	0.79		1.06 (0.88–1.27)	0.52
MRSA	41	136	2.40 (1.56–3.69)	<0.001		1.54 (1.06–2.34)	0.026		1.38 (0.88–2.17)	0.15
MRSA SCC*mec* II	36	83	3.38 (2.00–5.72)	<0.001		1.42 (0.88–2.26)	0.14		1.40 (0.87–2.24)	0.16
MRSA SCC*mec* IV	5	53	1.07 (0.37–3.12)	0.9		2.05 (0.60–7.03)	0.25		1.96 (0.55–6.92)	0.29
In colonized participants										
MSSA	279	2,668	1 (referent)			1 (referent)			1 (referent)	
MRSA	41	136	2.90 (1.92–4.40)	<0.001		1.48 (0.94–2.32)	0.09		1.27 (0.76–2.13)	0.35
MRSA SCC*mec* II	36	83	4.05 (2.42–6.79)	<0.001		1.36 (0.84–2.21)	0.2		1.17 (0.66–2.08)	0.58
MRSA SCC*mec* IV	5	53	1.30 (0.44–3.84)	0.63		1.90 (0.54–6.64)	0.3		1.97 (0.58–6.70)	0.26

*HR, hazard ratio; MSSA, methicillin-susceptible *S. aureus*; MRSA, methicillin-resistant *S. aureus*; SCC*mec*: staphylococcal cassette chromosome *mec*. †Generated by using Cox proportional hazards regression. ‡Categorized into no *S. aureus* colonization (0), MSSA colonization (1) and MRSA colonization (2), to generate hazard ratios for MSSA colonization (1) and MRSA colonization (2) in reference to no *S. aureus* colonization (0). In the analysis by SCC*mec* types, exposure was categorized into: no *S. aureus* colonization (0), MSSA colonization (1), MRSA SCC*mec* II (2), and MRSA SCC*mec* IV (3) and hazard ratios for MRSA SCC*mec* II (2), and MRSA SCC*mec* IV were reported. In colonized participants, the analysis was repeated with MSSA colonization being the reference category. §Adjusted for age, sex, race/ethnicity, and Poverty Income Ratio. ¶Adjusted for age, sex, race/ethnicity, poverty Income ratio, smoking, body mass index, and co-morbidities (asthma, diabetes, hypertension, cardiovascular diseases, stroke, liver disease, and cancer).

Likewise, colonization with SCC*mec* type II MRSA more strongly predicted long-term mortality than did no *S. aureus* ((HR 3.38, 95% CI 2.00–5.72) or MSSA colonization (HR 4.05, 95% CI 2.42–6.79) in univariate analyses but not after adjustment for sociodemographic characteristics. Colonization with SCC*mec* type IV MRSA was not associated with death in univariate or adjusted analysis, unlike no *S. aureus* or MSSA colonization (Table 2). However, the small number of participants with MRSA SCC*mec* types II (83) and IV (53) limited our analysis.

## Conclusions

In this population-based study, we found that MSSA is not associated with long-term mortality. MRSA is associated with risk for death in crude analysis but not after adjustment for socioeconomic factors, co-morbidities, or both.

Regarding MSSA, our results are consistent with studies conducted in inpatient and nursing home participants that also failed to find an association (*4*–*7*). Many of these studies were underpowered, calling for larger scale investigations. In line with the inverse association in our unadjusted analysis for MSSA and mortality, Wertheim et al. found that MSSA-colonized participants had longer survival than did noncolonized participants among patients with *S. aureus* bacteremia (*3*). They suggested that colonized patients might be more immunologically adapted to the endogenous *S. aureus* strains that caused the bacteremia or that exogenous strains in noncolonized patients were more virulent. In our case, we hypothesized that the negative association was imputable to some confounders, such as race/ethnicity, smoking, and co-morbidities (hypertension, cardiovascular diseases, stroke), instead of an independent association. We found that persons with MSSA were more likely to be non-Hispanic white and have asthma or diabetes than were persons without *S. aureus* nasal carriage, who were more likely be non-Hispanic black; have a lower poverty income ratio; be a smoker; and have hypertension, cardiovascular diseases, stroke, liver disease, or cancer. Concerning MRSA, Chan et al. observed that MRSA colonization was associated with higher mortality than non-MRSA colonization in nursing home residents in China followed for 2 years (*8*). Previous research also found that methicillin resistance in *S. aureus* bacteremia did not predict long-term mortality after adjustment for age, comorbidities, severity of acute illness, metastatic infections, and long-term facility resident status (*1*).

Delayed treatment initiation, virulence factors such as PVL, or both have been hypothesized to be responsible for excess mortality in MRSA infections and bacteremia. Subsequent research found that PVL was associated with decreased time from colonization to bacteremia but not to mortality (*9*). Again in confirmation of our results, Han et al. studied the effect of SCC*mec* type on the 30-day in-hospital mortality in 184 patients with MRSA and found SCC*mec* II significant in univariate analysis, but not after co-variates adjustment (*10*). That study also concluded that SCC*mec* II was a marker for disease severity instead of a predictor of mortality. 

Our study had several limitations. The carriage of *S. aureus* could be transient in some cases and not always reflect the carriage pattern over time. Although the nares are the most common site for *S. aureus* carriage, extranasal sites can also harbor the organism (*11*). *S. aureus* carriage was tested in 2001–2004 and might not reflect the current epidemiology and MRSA strain types. Data on co-morbidities, such as cardiovascular or liver diseases and cancer, were not available for participants 18–19 years of age and were coded as missing in the analyses. We did not compare death for the genes encoding enterotoxins because of their limited numbers. However, these NHANES data are the only national data available, and the major strengths of our study include the sample size representative of the US population with generalizable findings and higher power for statistical inferences.

Our findings are of public health and clinical importance because a lack of independent association between colonization by *S. aureus* and mortality implies that decolonization might not prevent death. Some studies indicate that decolonization of patients at high risk for infections could reduce the number of subsequent *S. aureus* infections, but evidence remains limited that it prevents death (*5*).
